# Supplementary material for: A Global Screen for Assembly State Changes of the Mitotic Proteome by SEC-SWATH-MS
Source: Cell Syst. 2020 Feb 26;10(2):133–155.e6. doi: 10.1016/j.cels.2020.01.001 (PMC7042714; doi:10.1016/j.cels.2020.01.001)

Q13405 | RM49_HUMAN | MRPL49 C11orf4 NOF1 OK/SW−cl.67
Monomer MW [kDa]: 19.198 Monomer expected elution fraction: 55

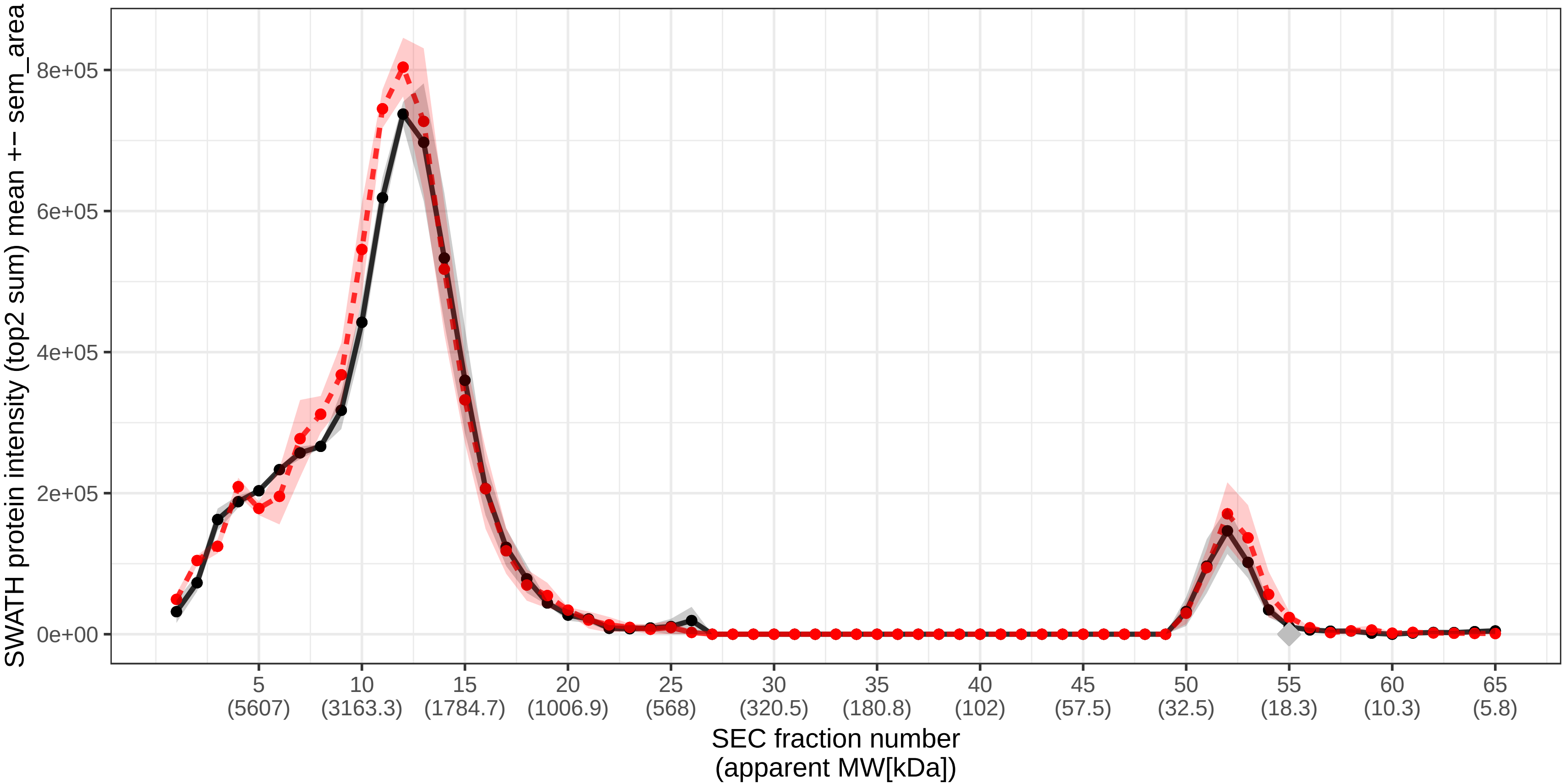

Supplement: Data S1. SEC-SWATH-MS Protein Chromatograms, Related to Figure 1 [file mmc6.zip › SECchrom_Q13405_RM49_HUMAN_MRPL49_C11orf4_NOF1_OK_SW-cl.67.pdf]
